# Anxiolytic and Antidepressant Effects of* Maerua angolensis *DC. Stem Bark Extract in Mice

**DOI:** 10.1155/2018/1537371

**Published:** 2018-09-09

**Authors:** Charles Kwaku Benneh, Robert Peter Biney, Donatus Wewura Adongo, Priscilla Kolibea Mante, Felix Agyei Ampadu, Augustine Tandoh, Jonathan Jato, Eric Woode

**Affiliations:** ^1^Department of Pharmacology, School of Pharmacy, University of Health and Allied Sciences, Ho, Ghana; ^2^Department of Pharmacology, School of Medical Sciences, University of Cape Coast, Cape Coast, Ghana; ^3^Department of Pharmacology, Kwame Nkrumah University of Science and Technology, Kumasi, Ghana; ^4^Department of Pharmacology, School of Pharmacy, Central University, Ghana; ^5^Department of Pharmacognosy and Herbal Medicine, University of Health and Allied Sciences, Ho, Ghana

## Abstract

**Introduction:**

The stem bark extract of* Maerua angolensis* DC. (Capparaceae) is used as a traditional remedy for management of anxiety, psychosis, and epilepsy.

**Aim of the Study:**

We therefore aimed at evaluating the anxiolytic and antidepressant potential of the plant in mice models.

**Methods:**

The dried stem bark was extracted with petroleum ether/ethyl acetate (50:50) mixture to obtain the extract, MAE. We employed Irwin's test to identify the preliminary behavioral and autonomic effects. Subsequently, MAE was administered* per os *to male mice and subsequently assessed, 1 h later, for anxiety parameters in the elevated plus maze (EPM) and the regular Suok tests. The forced swim (FST) and tail suspension (TST) tests were employed to assess the antidepressant potential of the extract (100-1000 mg kg^−1^).

**Results:**

In our preliminary assay, MAE (100-5000 mg/kg) exhibited analgesic effects and a reduction in fear response in the Irwin's test. The spontaneous locomotor activity was reduced at 1000 mg/kg. Additionally, MAE (1000 mg/kg) increased the latency to PTZ-induced convulsions, and duration to sleep in the pentobarbitone induced sleeping time assay. MAE (1000 mg/kg), similar to diazepam, in the anxiolytic assay, increased the percentage time spent in the open arms while decreasing protected head dips and unprotected stretch attend postures in the EPM. Correspondingly, there was a reduction in anxiety-induced immobility and freezing in the Suok test (300 mg/kg) without loss of sensorimotor coordination. Additionally, there was a significant reduction in immobility duration in the FST (300 mg/kg) and TST (1000 mg/kg).

**Conclusion:**

The petroleum ether/ethyl acetate fractions of* Maerua angolensis* stem bark possess anxiolytic and acute antidepressant effects in mice.

## 1. Introduction

Depression is a significant contributor to the total economic and health burden of every country [1]. This burden is more severe in third world countries where diagnosis and medications for treatment are inadequate and relatively expensive [2]. In contrast, most current treatment regimen available have proven less efficacious at ameliorating the condition. Anxiety is usually comorbid with depression states and treatment options that assuage both conditions are associated with a higher efficacy with a correspondingly lower relapse rates [3]. This has made the search for molecules with superior pharmacological profile and possibly effective at multiple related targets important.

Plants have served as a rich source of new molecules with pharmacological properties that fill an essential gap in the search for superior therapeutic agents. Local remedies, over the years, have served as a relatively cheap source of therapy and have been employed in the management of disorders such as anxiety, schizophrenia, and epilepsy. The therapeutic claims of preparations from local herbs have over the years provided valuable clues for the direction of pharmacological investigations [4–6]

*Maerua angolensis* DC. (Capparaceae) is a local plant found in various parts of West and Central Africa with a myriad of uses for neurologic disorders [7]. The root and stem bark decoction is sedating and have been used in the management of pain, epilepsy and psychosis [7–10]. Additionally,* Maerua angolensis* is used in traditional medicine for ameliorating anxiety associated with other disease states such as schizophrenia. Recent pharmacological investigations demonstrate that the plant possesses significant* in vivo* antioxidant [11] and anti-inflammatory [8, 12] properties.

Despite the plants popular use, there is sparse scientific evidence supporting its purported CNS activity. Hence, it is important to investigate the potential of* Maerua angolensis *extract in anxiety and the related disorder depression in order to provide some scientific evidence for the plants folkloric use. The current work assessed the anxiolytic potential of* Maerua angolensis* extract in the elevated plus maze, open field, and Suok tests in mice. We further explored the potential antidepressant effects of* Maerua angolensis* extract in the tail suspension and forced swim tests.

## 2. Methods

### 2.1. Plant Extraction and FT-IR Analysis of Crude Extract

*Maerua angolensis *extract was obtained according to methods described by Benneh and colleagues [13, 14]. The concentrate obtained was further dried in a hot air oven at 55°C for 72 h to obtain a green semisolid mass (~8.5 g) which was then stored in the freezer at -40°C until use.

The spectral region between 400 and 1400 cm^−1^ is usually considered as the unique region for every compound/compound mixtures and hence can be used for identification and quality control. Hence, triplicate FT-IR (PerkinElmer® UATR Two) spectra were subsequently generated for the extract.

#### 2.1.1. Chemicals and Drugs

Imipramine; pentylenetetrazole; caffeine; sodium pentobarbitone; Tween 80 (Sigma-Aldrich Inc., St. Louis, MO, USA), fluoxetine (Eli Lilly and Co., Indianapolis, IN, USA), diazepam (INTAS, Gujarat, India) were used. Caffeine, pentobarbitone, diazepam, and pentylenetetrazole were dissolved in distilled water before oral or intraperitoneal administration. To avoid temperature-induced breakdown of pentylenetetrazole, the solution was constantly kept on ice throughout the experimental duration.* Maerua angolensis *extract (MAE), fluoxetine, and imipramine were prepared by solubilizing the fine powder with Tween 80* q.s.* A maximum of 1 mL was delivered by oral gavage for* per os *treatment. A maximum volume of 0.2 mL was set for subcutaneous injection and 1.0 mL for intraperitoneal injection.

### 2.2. Animals

Male ICR mice (20-25 g) were obtained from the vivarium of the Department of Pharmacology, KNUST, Kumasi, Ghana. They were housed, in groups of 5, in stainless steel cages (34 × 47 × 18 cm^3^) with soft wood shavings as bedding and housing conditions as follows: temperature maintained at 23-25°C, relative humidity 60-70 %, and 12 h light-dark cycle. All mice had free access to water and pellet diet (GAFCO, Tema, Ghana). All experiments were compliant with NIH Guidelines for the Care and Use of Laboratory Animals. Ethical approval was obtained from the Department of Pharmacology, Animal Ethics Committee, KNUST..

### 2.3. Preliminary Neuropharmacological Screening

#### 2.3.1. Irwin Test

Irwin's test is a battery of tests that assesses the behaviour and autonomic response of mice/rats after pretreatment with a compound. The test measures individual behavioural and autonomic parameters of each mouse in a regularly spaced time interval. In addition to the parameters assessed the test provided data on the lethality of the extract or compound in a 24 h period.

Mice were randomly distributed into seven groups (n=5) and left to acclimate to the experimental room for 24 h. Mice were fasted during this period but had free access to glucose solution. The animals were treated with six (6) oral doses of MAE (30, 100, 300, 1000, 3000, and 5000 mg kg^−1^* p.o.*) while the control group received 1% tween in distilled water (10 ml kg^−1^* p. o.*). The neurologic, autonomic, and behavioural state of the animals were observed at 0 to 15, 30, 60, 120, 180 min, 24 h, and 48 h as described by Irwin [15].

#### 2.3.2. Activity Meter Test

The effect of MAE on spontaneous locomotor activity was evaluated using an activity cage (Ugo Basile model 7401, Comerio, VA, Italy). Mice (20-25 g) were randomly divided into eleven groups (n=6-8) and treated with MAE (30, 100, 300, 1000 mg kg^−1^* p.o.),* diazepam (0.1, 0.3, 1.0 mg kg^−1^ i.p.), caffeine (10, 30, 100 mg kg^−1^* p.o*.), or 1% tween in distilled water (10 ml kg^−1^* p.o.*). Animals were then placed individually in the activity cage and their activity scored in 5 min blocks for 30 min.

#### 2.3.3. Convulsive Threshold Test

Male mice randomly were assigned to six (6) groups and received either MAE (30, 100, 300, 1000 mg kg^−1^* p.o*.), diazepam (8 mg kg^−1^ i.p.), or 1% tween in distilled water (10 ml kg^−1^* p.o*.). One hour after oral or 30 minutes after i.p. treatment, seizure was induced by subcutaneous administration of pentylenetetrazole (85 mg kg^−1^), at the nape of the neck, and immediately placed in plastic observational cages. The seizure characteristics after PTZ injection were recorded for thirty minutes with the aid of a camcorder. The video output was then analyzed for the latency to clonic seizure and number of death with the behavioural analysis software, JWatcher® version 1.0 (University of California, Los Angeles, USA and Macquarie University, Sydney, Australia. Available at http://www.jwatcher.ucla.edu/).

#### 2.3.4. Pentobarbitone Induced Sleeping Time Test

The effect of MAE on pentobarbitone-induced sleeping time was investigated. Male mice were assigned randomly to seven groups (n=7) and received either MAE (30, 100, 3000, and 1000 mg kg^−1^* p.o*.), diazepam (8 mg kg^−1^* p.o*.), caffeine (16 mg kg^−1^* p.o*.), or 1% tween in distilled water (10 ml kg^−1^* p.o*.). Sodium pentobarbitone (50 mg kg^−1^ i.p) was administered 1 h after respective drug treatments. Latency to sleep (time between pentobarbitone injection and loss of righting reflex) and duration of sleep (time between loss of and regaining of righting reflex) were recorded with a stopwatch.

### 2.4. Anxiety Screening

#### 2.4.1. Regular Suok Test

The regular Suok test was carried out as described by Kalueff et al., 2008, with slight modifications [16]. The setup consisted of a 2-meter aluminum rod (diameter = 2 cm) divided into 10 equal segments and elevated 25 cm high. To avoid or reduce harm to mice falling from the rod, the base of the setup was covered with a thick layer of paper towels.

Forty-two (42) mice were allowed to acclimatize for 24 h in a dimly lit experimental room for an hour before drug treatment and testing. Mice were the randomly selected and distributed into seven groups of six (6) animals each. Animals received either MAE (30, 100, and 300 mg kg^−1^,* p.o*.), diazepam (0.1, 0.3, and 1.0 mg kg^−1^, i.p.), or 1% tween in distilled water (10 ml kg^−1^,* p.o*.). One hour after oral and thirty minutes after intraperitoneal administration, mice were placed in the central region of the rod. The behaviour on the rod was captured for five (5) minutes with the aid of a camcorder mounted approximately 2 meters away from the rod. The exploratory activity and specific behaviours were then scored and analyzed with the aid of JWatcher software. Behaviours assessed included (a) duration of immobility, (b) frequency of freezing, and (c) number of leg slips. Mice that fell off the rod were returned to the position of fall and recording continued.

#### 2.4.2. Elevated Plus-Maze

The elevated plus-maze test was performed according methods described by to Pawlak et al. [17]. The elevated plus maze consists of two closed (30 × 5 × 1 cm^3^) and two open arms (30 × 5 × 30 cm^3^) with a central arena (5 × 5 cm^2^). The maze is elevated 60 cm above the floor with the aid of a platform. Behavioural testing was performed under dim light in a noise-attenuated room. Fifty-six (56) ICR mice were randomly selected and distributed into ten groups of seven (7) animals each. Animals received either MAE (30, 100,300, and 1000 mg kg^−1^,* p.o*.), diazepam (0.1, 0.3, and 1.0 mg kg^−1^, i.p.), or 1% tween in distilled water (10 ml kg^−1^,* p.o*.). The maze was cleaned with 10 % v/v ethanol after each session to remove olfactory cues. With the aid of camcorder and the JWatcher software, the following behaviours were recorded and scored: (i) number of closed and open arm entries, (ii) time spent in exploring open and closed arms, (iii) frequency and duration of protected and unprotected head dips (leaning over the edge of the open arms), and (iv) frequency and duration of protected and unprotected stretch attend postures (mouse stretches forward and retracts without moving its feet). An arm entry was defined as a mouse having entered one arm of the maze with all four limbs. Behaviours were defined as protected if they occur in the closed arms or center and unprotected when they were exhibited in the open arm region of the maze.

### 2.5. Acute Antidepressant Tests

#### 2.5.1. Forced Swim Test

The forced swim test was carried out according to the method described by Porsolt et al. [18]. Seventy (70) ICR mice were randomly assigned to ten groups of seven animals each. After acclimatization, mice were dosed with either MAE (100, 300, and 1000 mg kg^−1^,* p.o*.), fluoxetine (3, 10, and 30 mg kg^−1^,* p.o.*), imipramine (10, 30, and 100 mg kg^−1^* p.o.*), or 1% tween in distilled water (10 ml kg^−1^* p.o*.) 60 minutes before behavioural testing. Mice were gently dropped individually into identical transparent cylindrical tanks (25 cm high and 10 cm deep) containing water (26 ±1°C) up to 20 cm for a total of 6 minutes. Each session was videotaped with a camcorder suspended above the cylinder. The duration of immobility, latency to immobility, climbing, and swimming during the last 4 minutes were quantified using JWatcher Version 1.0®. After the end of each session, animals were removed from the cylinders dried with a towel and placed near a heater until they were completely dry. The latency to and duration of immobility give an indication of antidepressant-like activity. An increased latency and reduced immobility are typically exhibited by antidepressant agents. The type and duration of the escape oriented behaviours (climbing and swimming) can be used to predict the possible mechanism(s) of action to the agent been tested.

#### 2.5.2. Tail Suspension Test

The tail suspension test was carried out according to the method previously described by Steru et al., 1985. ICR mice were randomly assigned to ten groups of seven animals each. After acclimatization, mice were dosed with either MAE (100, 300, and 1000 mg kg^−1^,* p.o.*), fluoxetine (3, 10, and 30 mg kg-^1^,* p.o*.), imipramine (10, 30, and 100 mg kg^−1^* p.o*.), or 1% tween in distilled water (10 ml kg^−1^* p.o*.). One hour after oral dosing animals were suspended at their tail (1 cm from the tip) with an adhesive tape on a horizontal bar raised 50 cm from a tabletop. Behaviours exhibited by the mice were recorded for a period of 6 min and subsequently analyzed for escape-oriented behaviours such as pedaling, curling, and swinging and immobility time and were quantified for the last 4 min of each session.

Behaviours assessed included the following: (1) immobility, a mouse was judged to be immobile when it hung by its tail without engaging in any active behaviour; (2) swinging, a mouse was judged to be swinging when it continuously moved its paws in the vertical position while keeping its body straight and/or it moved its body from side to side; (3) curling, a mouse was judged to be curling when it engaged in active twisting movements of the entire body; (4) pedaling was defined as when the animal moved its paws continuously without moving its body. Mice that climbed on their tail were gently pulled down and the test continued.

### 2.6. Statistics

Data are presented as mean ± SEM. Data were analyzed using one-way analysis of variance (ANOVA). When ANOVA was significant, multiple comparisons between treatments were done using Sidak* post hoc *test.

Dose-response curves are constructed using iterative curve fitting with the following nonlinear regression (three-parameter logistic) equation:

(1) $$\begin{array}{c} Y=\frac{a+\left(b-a\right)}{\left({1+10}^{\left({\log ED}_{50}-X\right)}\right)} \end{array}$$

where* X* is the logarithm of dose and* Y *is the response.* Y* starts at* a* (the bottom) and goes to* b *(the top) with a sigmoid shape. The fitted midpoints (ED_50_) of the curves were compared statistically using* F* test with GraphPad Prism for Windows version 6.01 (GraphPad® Software, San Diego, CA, USA).

## 3. Results

### 3.1. FT-IR Analysis of Crude Extract

**Figure 1:**
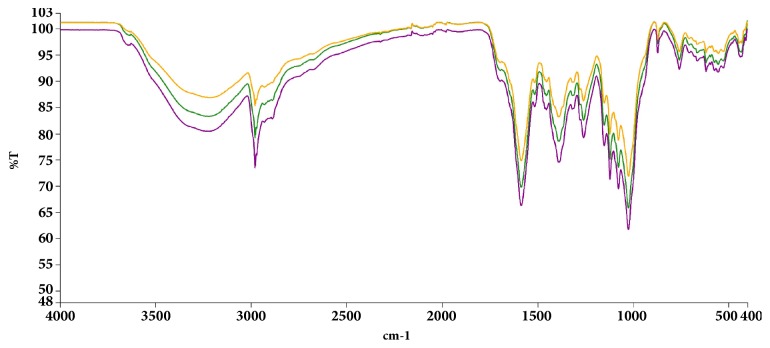
Baseline corrected infrared spectra of the petroleum ether/ethyl acetate fraction of* Maerua angolensis* stem bark extract. Experiment was repeated thrice, with similar conditions, from 400-4000 cm^−1^. Peak values and labels are available in the Appendix. [13, 14].

The characteristic spectra (Figure 1) in the region from 400 to 1400 cm^−1^ were as a fingerprint region for subsequent comparison of future extracts.

### 3.2. Preliminary Neuropharmacological Tests

#### 3.2.1. Irwin's Test

Oral administration of MAE produced analgesia and reduced fear response at all tested doses. The onset of action of these responses was observed to be shorter at higher doses (Table 1). Reduced touch response and increased jumps (lasting ~15 min) were also recorded after administration of 3000 and 5000 mg kg^−1^, respectively. MAE (100-5000 mg kg^−1^* p.o*.) administration did not have any lethal effects over the 48 h observation period.

#### 3.2.2. Activity Meter Test

**Figure 2:**
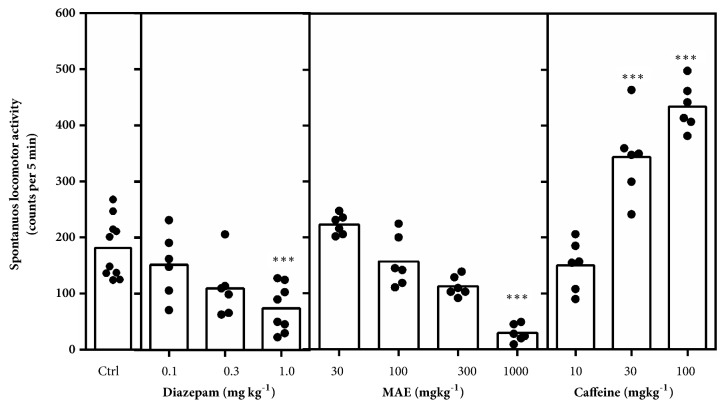
Effects of MAE (30-1000 mg kg^−1^,* p.o*.), diazepam (0.1-1.0 mg kg^−1^, i.p.), and caffeine (10-100 mg kg^−1^,* p.o*.) in the activity meter test. Significantly different from control: *∗∗∗P* < 0.001 (one-way ANOVA followed by Sidak* post hoc* test).

*Maerua angolensis *stem bark extract (*P*<0.0001) and diazepam (*P*<0.0001) at doses of 1000 mg kg^−1^ and 1.0 mg kg^−1^, respectively, reduced spontaneous locomotor activity (Figure 2). Caffeine (*P*<0.0001) on the other hand significantly increased the locomotor at 30 and 100 mg kg^−1^.

#### 3.2.3. Convulsive Threshold Test

**Figure 3:**
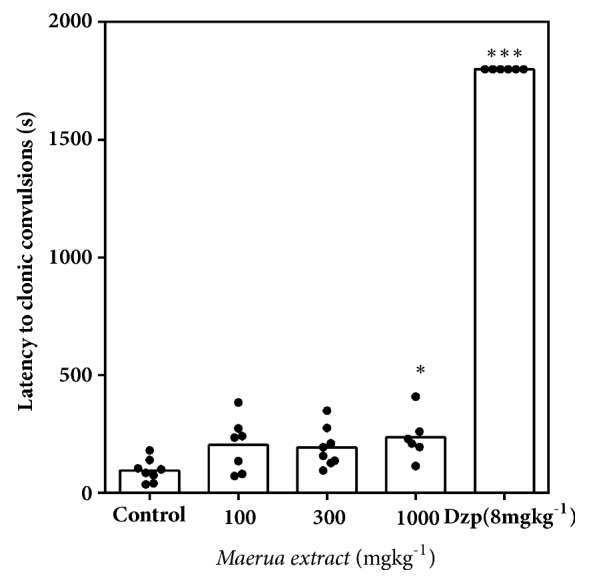
Effect of MAE (100- 1000 mg kg^−1^* p.o*.) and diazepam (8 mg kg^−1^* p.o*.) on the latency to clonic seizures in mice. Significantly different from control: *∗P*<0.05, *∗∗∗P*<0.001 (one-way ANOVA followed by Sidak* post hoc* test).

**Figure 4:**
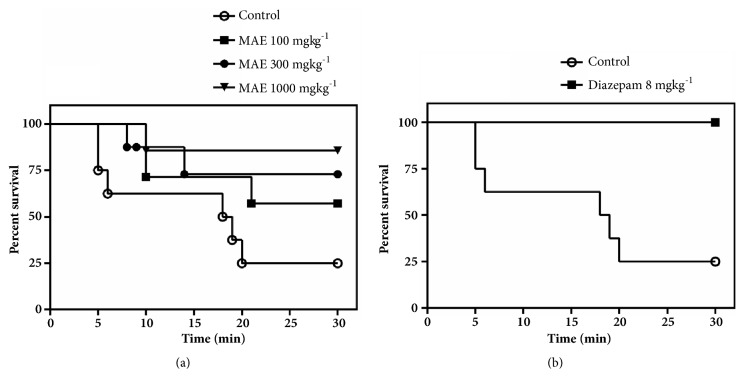
A Kaplan-Meier estimate of overall survival of animals treated with (a) MAE (100 -1000 mg kg^−1^* p.o*.) and (b) diazepam (8 mg kg^−1^* p.o*.) in the pentylenetetrazole-induced seizure test over a 30-min observation period.

MAE (1000 mg kg^−1^* p.o*.) delayed onset of PTZ-induced convulsions significantly (*P*<0.05, Figure 3). However due to lethality recorded before the 30th minute in the solvent control and MAE treated groups, the frequency and duration of convulsions were not assessed. A survival analysis (Figure 4) was rather employed to reveal the degree of protection offered by the administered agents. Although the survival analysis revealed a significant trend in the degree of protection offered by MAE, comparison with the solvent control, it did not reveal any significant statistical difference. Diazepam (8 mg kg^−1^), the reference anticonvulsant, increased the latency to convulsions (*P*<0.0001) while decreasing the lethality significantly.

#### 3.2.4. Pentobarbitone Induced Sleeping Time

**Figure 5:**
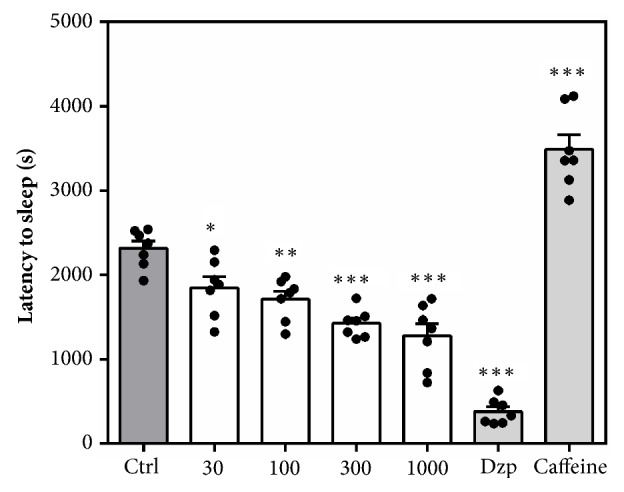
Effects of acute MAE (30-1000 mg kg^−1^ p.o.), diazepam (8 mg kg^−1^* p.o*.), and caffeine (16 mg kg^−1^ p.o.) on latency to sleep in the pentobarbitone-induced sleeping time. Data are presented as group mean ± SEM. Significantly different from control: *∗P*<0.05, *∗∗P*<0.01. *∗∗∗P*<0.001.

**Figure 6:**
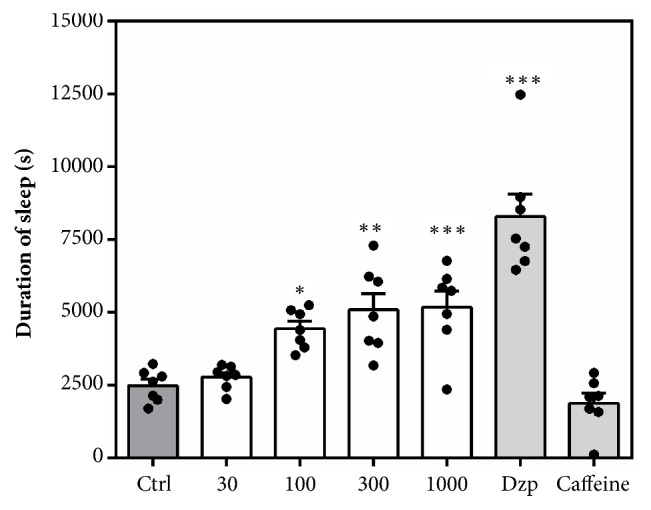
Effects of acute MAE (30-1000 mg kg^−1^,* p.o*.), diazepam (8 mg kg^−1^* p.o*.), and caffeine (16 mg kg^−1^* p.o*.) on sleep duration in the pentobarbitone-induced sleeping time. Data are presented as group mean ± SEM. Significantly different from control: *∗P*<0.05, *∗∗P*<0.01. *∗∗∗P*<0.001.

Acute administration of MAE (30 -1000 mg kg^−1^* p.o*.) at all doses exhibited a sedative potential after pretreatment with pentobarbitone. There was a dose-dependent and significant decrease (*F*_6,  42_= 71.06,* P *< 0.0001) in latency to loss of righting reflex (Figure 5). Diazepam (8 mg kg^−1^) and caffeine (16 mg kg^−1^) significantly decreased and increased the latency to sleep, respectively.  Figure 6 also shows a significant increase in duration of sleep after MAE (*F*_6,  42_ = 71.06* P* < 0.0001) administration. In contrast, the CNS stimulant (caffeine) did not significantly reduce the duration of sleep as compared to pentobarbitone control group.

### 3.3. Anxiety Tests

#### 3.3.1. Regular Suok Test

**Figure 7:**
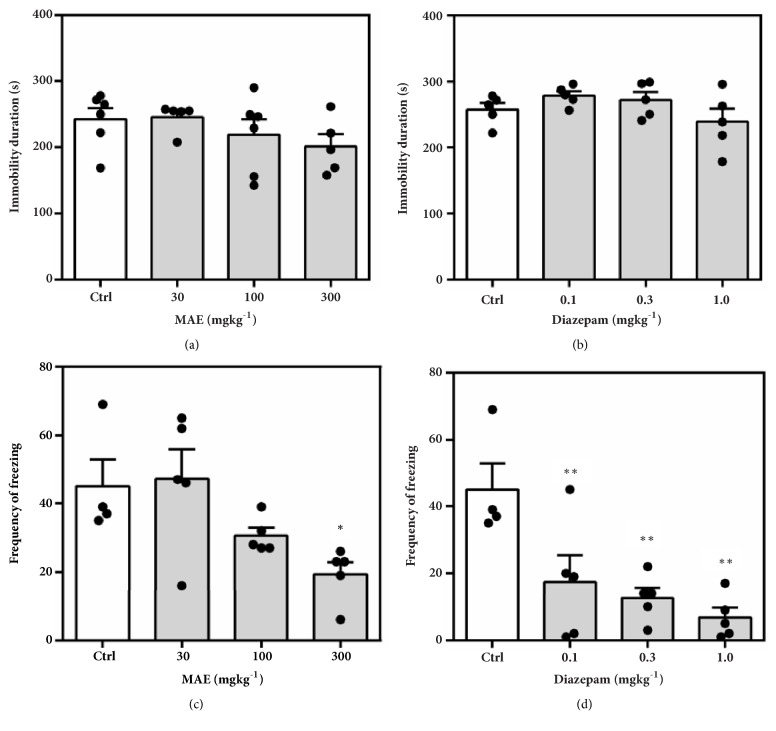
Effects of MAE (30-300 mg kg^−1^) and diazepam (0.1-1.0 mg kg^−1^) on duration of immobility (a & b) and number of freezing bouts (c & d) over a 5-min test period in the regular Suok test. Data are expressed as group mean ± SEM. Significant difference: *∗P*<0.05, *∗∗P*<0.01, *∗∗∗P*<0.001 compared to control group (one-way ANOVA followed by Sidak* post hoc* test).

**Figure 8:**
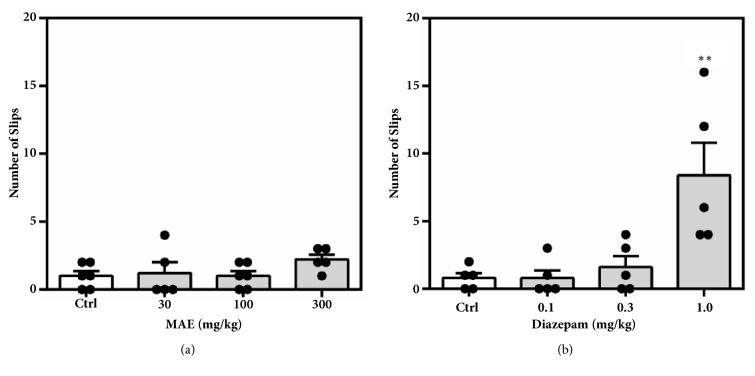
Effects of MAE (30-300 mg kg^−1^) and diazepam (0.1-1.0 mg kg^−1^) on the number of leg slips over a 5-min test period in the regular Suok test. Data are expressed as group mean ± SEM. Significant difference: *∗P*<0.05, *∗∗P*<0.01, *∗∗∗P*<0.001 compared to control group (one-way ANOVA followed by Sidak* post hoc* test).

Behaviours assessed include the following: duration and number of freezing bouts and leg slips. Although the duration of freezing (MAE= *F*_3,  17_ = 1.827* P*=0.1829, Dzp = *F*_3,  18_ = 1.220* P*=0.3311) was not significantly altered, the frequency of freezing bouts was significantly reduced at all dose levels of diazepam (*F*_3,  17_ = 7.59* P*=0.0026) and 300 mg kg^−1^ of MAE (*F*_3,  18_ = 4.653* P*=0.0172) (Figure 7). The number of leg slips, which gives an indication of the degree of locomotor impairment, was not significantly higher in mice treated with MAE compared to solvent treated (*F*_3,  18_ = 1.315* P*=0.3003). In contrast, there was a significant increase (*F*_3,  17_ = 7.876* P*=0.0019) in number of leg slips when mice were treated with 1.0 mg kg^−1^ of diazepam (Figure 8).

#### 3.3.2. Elevated Plus Maze

**Figure 9:**
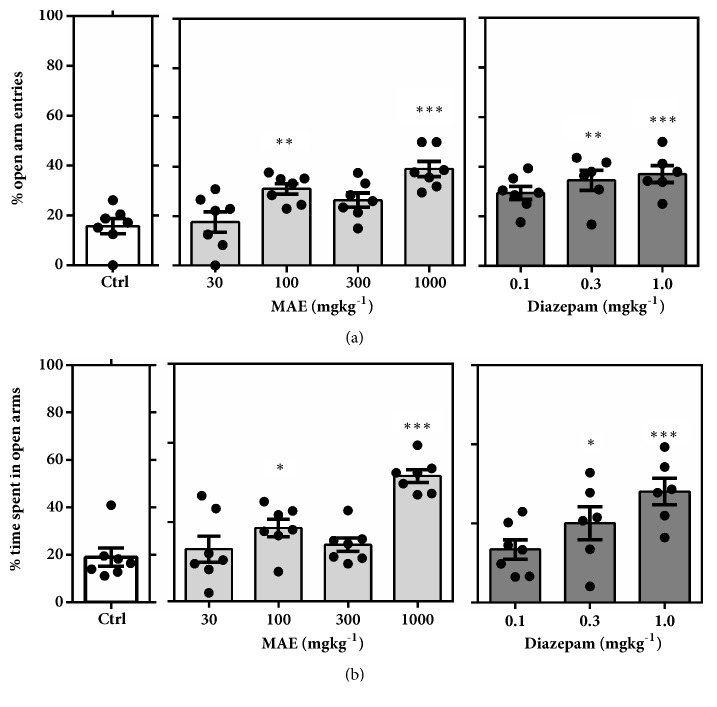
Effect of MAE (30-1000 mg kg^−1^) and diazepam (0.1-1.0 mg kg^−1^) on mice behaviour in the EPM test, over a 5-min test period. (a) Percentage open arm entries. (b) Percentage of time spent in the open arms of the EPM. Data are presented as group mean ± SEM. Significantly different from control: *∗P*<0.05, *∗∗P*<0.01, *∗∗∗P*<0.01 (one-way ANOVA followed Sidak* post hoc* test).

**Figure 10:**
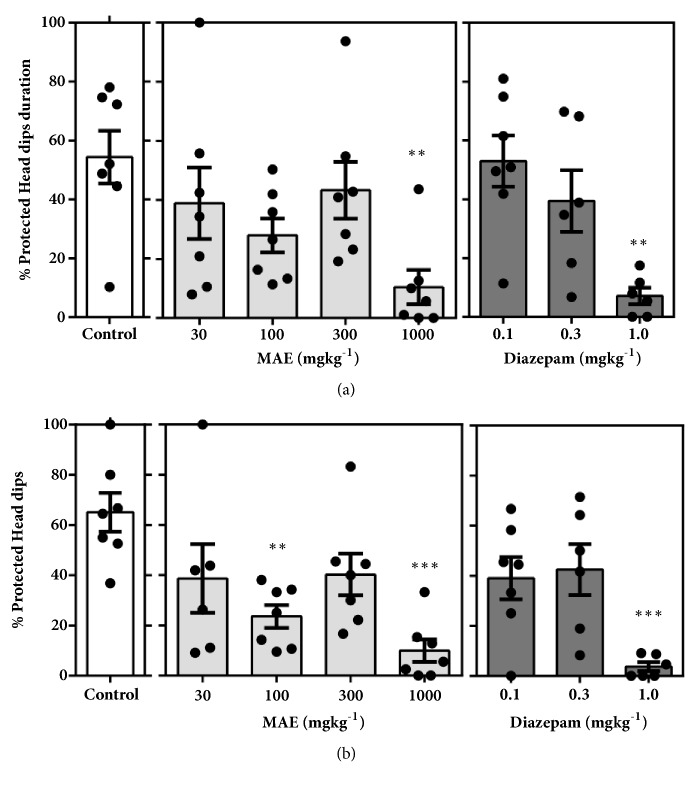
Effect of MAE (30-1000 mg kg^−1^) and diazepam (0.1-0.3 mg kg^−1^) on mice behaviour in the EPM test, over a 5-min test period. (a) Percentage duration of protected head dips. (b) Percentage of a number of protected head dips. Data are presented as group mean ± SEM. Significantly different from control: *∗∗P*<0.01, *∗∗∗P*<0.01 (one-way ANOVA followed Sidak* post hoc* test).

**Figure 11:**
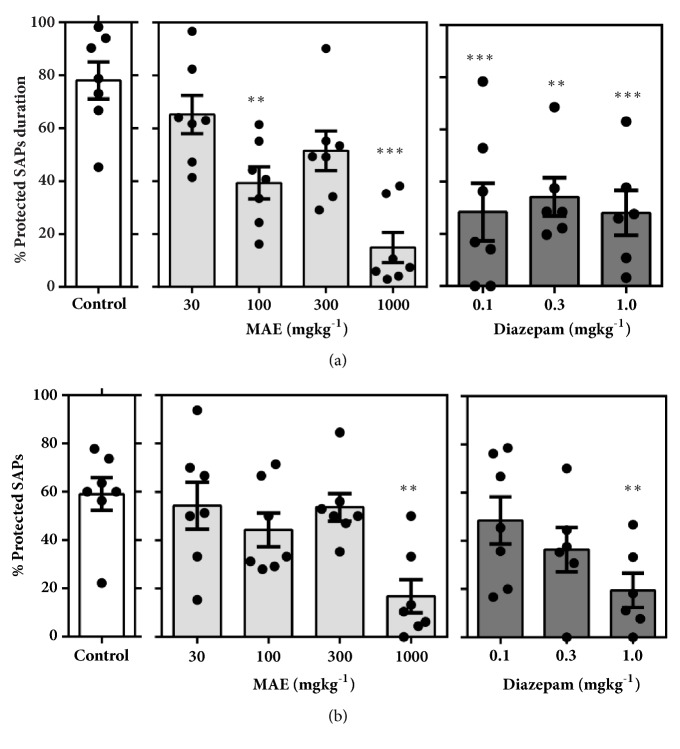
Effect of MAE (30-1000 mg kg-1) and diazepam (0.1-0.3 mg kg-1) on mice behaviour in the EPM test, over a 5-min test period. (a) Percentage duration of protected stretch attend postures (SAPs). (b) Percentage of number of protected SAPs. Data are presented as group mean ± SEM. Significantly different from control: *∗∗P*<0.01, *∗∗∗P*<0.01 (one-way ANOVA followed Sidak post hoc test).

Testing in the elevated plus maze after acute MAE administration significantly favoured anxiolytic parameters (Figures 9, 10, and 11). Figures 9(a) and 9(b) show a significant increase in % open arm entries (*F*_7,  46_ =7.212* P*<0.0001) and % time spent in the open arms (*F*_7,  46_ =9.718,* P*<0.0001). This significant increase in open arm exploration was observed after treatment with MAE (100 & 1000 mg kg^−1^) and diazepam (0.3 & 1.0 mg kg^−1^). Additionally, these doses produced a significant decrease in duration of protected head dips (*F*_7,  46_ =4.252,* P=*0.0011) and protected stretch-attend postures (*F*_7,  46_ =7.653,* P*<0.0001) (Figures 10(a) and 10(b)). The number of protected head dips (*F*_7,  45_ =6.152* P*<0.0001) and protected stretch attend postures (*F*_7,  46_ =4.091,* P=*0.0014), as a percentage, decreased in a similar fashion as seen above (Figures 10(b), 11(b)).

### 3.4. Acute Antidepressant Tests

#### 3.4.1. Forced Swim Test

**Figure 12:**
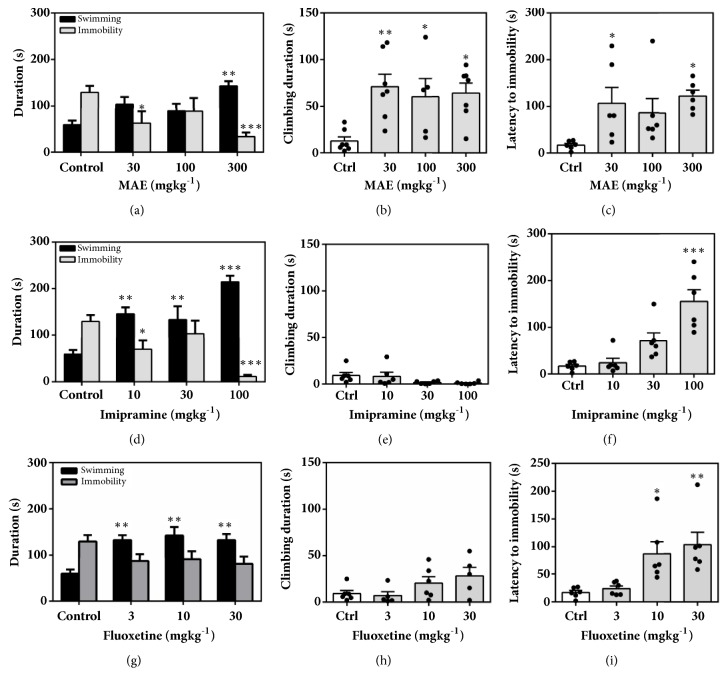
Performance of mice in the FST: behavioural assessment including immobility and swimming duration (a, d, g), climbing duration (b, e, h), and latency to immobility (c, f, i) after acute treatment with MAE (100-1000 mg kg^−1^), imipramine (10-100 mg kg^−1^), and fluoxetine (3-30 mg kg^−1^). Data are expressed as group mean ± SEM. Significantly different from control: *∗P*<0.05; *∗∗P*<0.01; *∗∗∗P* <0.001 (one-way ANOVA followed by Sidak* post hoc* test).

Figure 12 represents the effect of acute administration of MAE (100-1000 mg kg^−1^* p.o.*), imipramine (10-100 mg kg^−1^*p.o.*), or fluoxetine (3-30 mg kg^−1^*p.o.*) on mice behaviours in the forced swim test.

**Figure 13:**
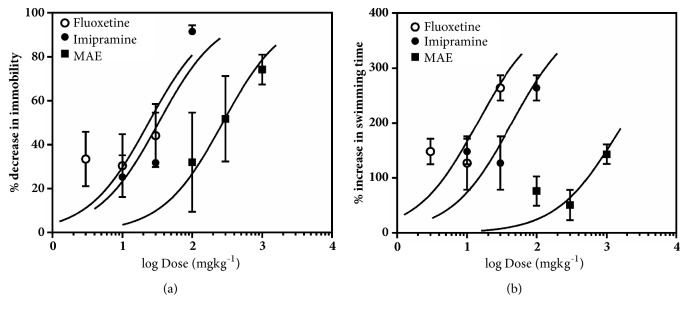
Dose-response curves for MAE (100-1000 mg kg^−1^), fluoxetine (3-30 mg kg^−1^), and imipramine (10-100 mg kg^−1^) with respect to (a) decrease in immobility and (b) increase in swimming time in the forced swim test. Each point represents the mean ± SEM (n=7).

From Figure 13, it was observed that oral administration of fluoxetine, MAE, and imipramine reduced immobility time in a dose-dependent manner by a maximum (E_max_) of 44.11 ± 14.29%, 71.72 ± 7.78%, and 91.47 ± 2.865%, respectively. The ED_50_ values show the order of potency of the test compounds as MAE <imipramine < fluoxetine.

A two-way ANOVA analysis revealed that 1000 mg kg^−1^ of MAE significantly decreased the immobility time and increased swimming time (*F*_3,  38_=10.33,* P*<0.0001) of mice in the FST (Figure 12(a)).* Post hoc* analysis revealed statistical significance for the effect of MAE on climbing (*F*_3,  22_=5.271,* P*=0.0068) at all tested doses and also increased the latency to immobility (*F*_3,  20_=3.718* P*=0.0283) at 100 and 1000 mg kg^−1^ of MAE (Figures 12(b) and 12(c)).

Similar to MAE, ANOVA analysis revealed that fluoxetine at all tested doses significantly increased swimming time (*F*_3,  40_=2.433,* P*=0.0002) of mice in the FST. The latency to immobility (*F*_3,  18_=8.209,* P*=0.0984) was significantly affected but not the climbing duration.

Imipramine, similar to the above treatment, increased the swimming time duration at all doses and decreased the immobility duration at 100 and 1000 mg kg^−1^ (*F*_3,  40_=19.67,* P<*0.0001). Although the duration of climbing (*F*_3,  20_=2.325,* P*=0.1056) was not affected, the latency to immobility (*F*_3,  20_=16.02,* P*<0.0001) was significantly increased after 100 mg kg^−1^ treatment with imipramine.

#### 3.4.2. Tail Suspension Test

**Figure 14:**
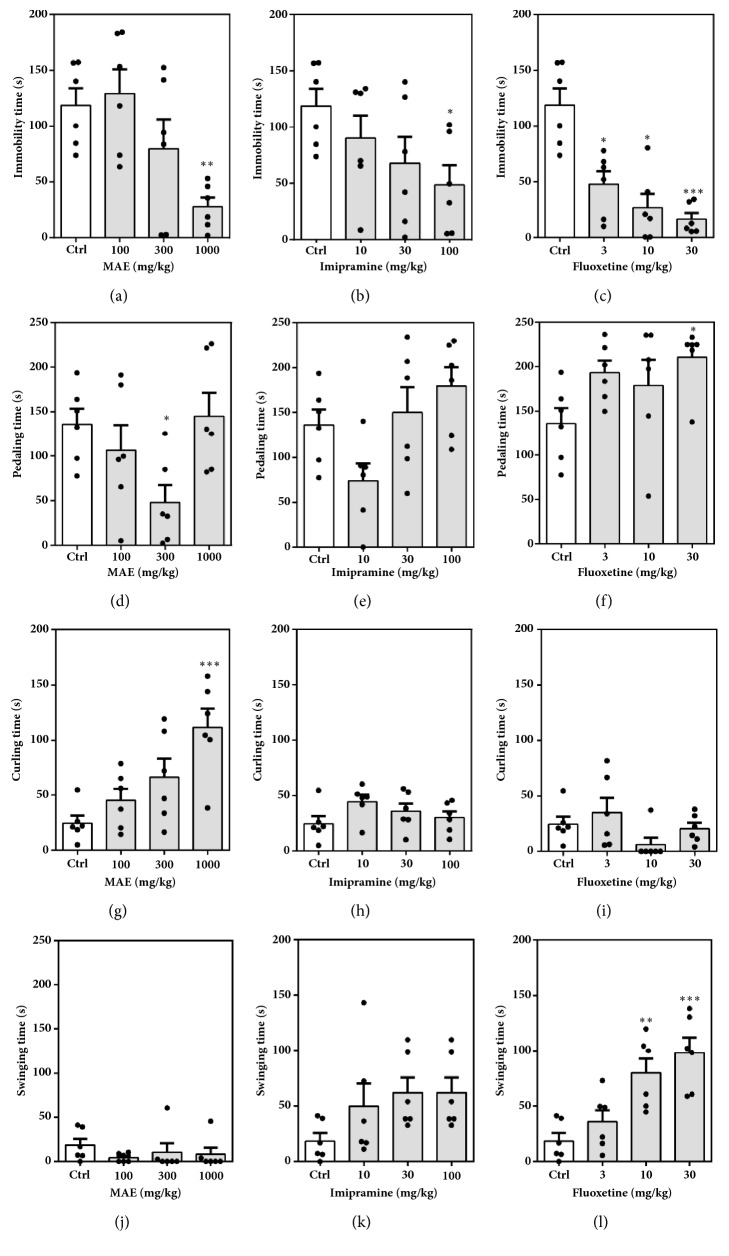
Performance of mice in the TST: behavioural assessment including duration of immobility (a, b, c), pedaling (d, e, f), curling (g, h, i), and swinging (j, k and l) after acute treatment of mice with MAE (100-1000 mg kg^−1^), imipramine (10-100 mg kg^−1^), and fluoxetine (3-30 mg kg^−1^). Significantly different from control: *∗P*<0.05; *∗∗P*<0.01; *∗∗∗P* <0.001 (one-way ANOVA followed by Sidak* post hoc* test).

Figure 14 represents the effect of acute administration of MAE (100-1000 mg kg^−1^* p.o.*), imipramine (10-100 mg kg^−1^), or fluoxetine (3-30 mg kg^−1^) on mice behaviours in the tail suspension test.

**Figure 15:**
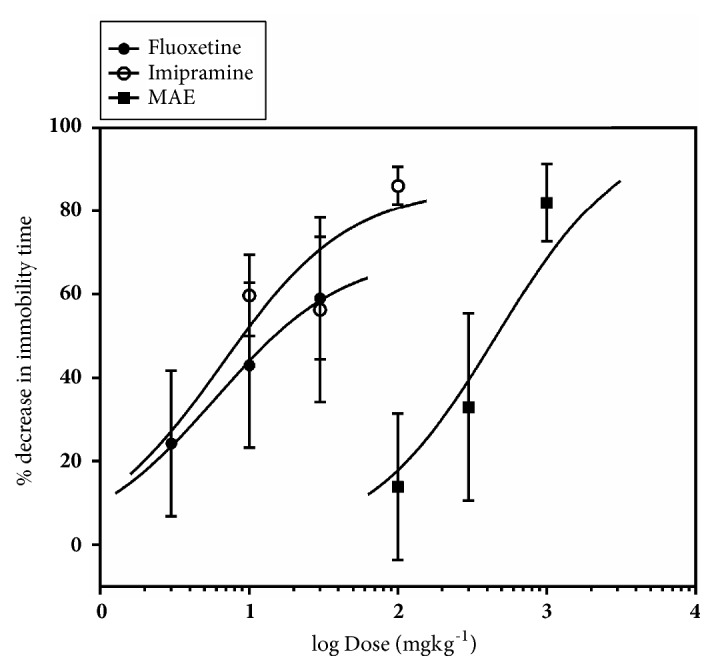
Dose-response curves for MAE (100-1000 mg kg^−1^), fluoxetine (3-30 mg kg^−1^), and imipramine (10-100 mg kg^−1^) with respect to % decrease in immobility in the tail suspension test in mice. Each point represents the mean ± SEM (n=7).

Administration of fluoxetine, MAE, and imipramine reduced immobility time in a dose-dependent manner by a maximum (E_max_) of 56.07 ± 14.62%, 82.06 ± 9.35%, and 86.19 ± 4.56%, respectively (Figure 15). The ED_50_ values show the order of potency of the test compounds: MAE < fluoxetine < imipramine.

Holm-Sidak* post hoc* test following one-way ANOVA test revealed the MAE at 1000 mg kg^−1^ significantly decreased the immobility time (*F*_3  20_ = 5.744,* P *= 0.0053) when compared to the control group. Similarly, imipramine at 100 mg kg^−1^ (*F*_3,  20_ = 2.412,* P* = 0.0969) and all test doses of fluoxetine (*F*_3,  20_ = 6.846,* P* = 0.0023) decreased immobility significantly.

The duration of pedaling was significantly altered after MAE (300 mg kg^−1^) (*F*_3,  20_ = 3.493,* P* = 0.0347) and fluoxetine (30 mg kg^−1^) (*F*_3,  20_= 2.681,* P* = 0.0745) but not imipramine treatment. MAE (*F*_3,  20_ = 0.6709,* P* = 0.5799) and imipramine (*F*_3,  20_ = 1.962* P* = 0.1523) treatment did not affect the swinging time; however, fluoxetine (*F*_3,  20_ = 10.68,* P* = 0.0002) at doses of 10 mg kg^−1^ and 30 mg kg^−1^ caused a significant increase. The cumulative duration of curling for the 5-min test period was significantly increased only after 1000 mg kg^−1^ MAE (*F*_3,  20_= 7.558,* P* = 0.0014) treatment.

## 4. Discussion

The current study demonstrates the anxiolytic and acute antidepressant effects of the petroleum/ethyl acetate extract of* Maerua angolensis *stem bark. Contrary to results obtained in other studies [9], the fraction we employed showed no significant antiseizure activity. The present study demonstrates that the lipophilic fraction of the stem bark extract demonstrates significant anxiolytic and antidepressant activity in male mice. These anxiolytic effects are in agreement with zebrafish anxiolytic and antidepressant studies [13].

The Irwin test evaluates the qualitative effects of test substances on the behaviour and autonomic and the physiological function of a test animal [15]. Results from such test can give an approximate onset and duration of action of different measured effects. In the Irwin test, the extract showed analgesic effect and reduction to fear and touch response at doses of 100-5000 mg kg^−1^* p.o.*

Continuous observation for 48 hours after the test revealed no physical signs of toxicity or lethality at all tested doses. This suggests that the LD_50_ in mice is beyond 5000 mg kg^−1^. The onset of action was increased with dose increments, with the fastest onset observed at 30 min. Based on onset and duration of the effects registered in the Irwin test, further tests were carried out 60 minutes after oral administration since lower doses (below 1000 mg kg^−1^) were adopted for subsequent tests.

The activity meter test was then employed to assess, quantitatively, the spontaneous behaviour with respect to locomotion after oral administration of MAE. There was significant reduction in locomotor activity after 1000 mg kg^−1^ MAE administration. Locomotor activity can be reduced significantly after dosing test animals with a sedative dose of CNS depressants. Also, a reduction in locomotor activity could also be due to motor impairment induced by the test compound. Consequently, the effects observed after MAE administration can be attributed to the sedative or locomotor impairment potential of the extract. Caffeine, a CNS stimulant, on the other hand, increased whilst diazepam, a CNS depressant, decreased the locomotor activity in this test.

In the Irwin test, test compounds that possess seizure induction potential can be identified by observing physical signs such as tonic and or clonic convulsions during the test. However, the test is not sensitive at identifying proconvulsant effects of compounds. Instead, the proconvulsant potential (seizure liability) is uncovered after pretreatment with chemoconvulsants such as pentylenetetrazole. Such treatments can additionally be used to screen potential anticonvulsants since compounds with anticonvulsant properties being known to reduce seizure parameters induced by these agents. In the convulsive threshold test in mice, only the highest dose of the MAE increased the latency to clonic convulsion and survival compared to the saline group. A Kaplan-Meier analysis of survival revealed no significant protection compared to vehicle control. This indicates that the extract was not effective in delaying and preventing lethality induced by pentylenetetrazole (85 mg kg^−1^, s.c.).

Barbiturates are general CNS depressants that induce a state of calm, sedation, and hypnosis at high doses [19]. In mice, acute administration of barbiturates induces a state of sleep which is indicated by a loss of righting reflex. Consequently, the sedating effects of potential CNS depressants are usually unmasked by coadministration with pentobarbitone. This sedative effect is known to be reversed by stimulants and enhanced general CNS depressants. A significant decrease in latency to sleep and increase in sleep duration after coadministration with pentobarbitone indicates that MAE possesses sedative effects, which is in agreement with results obtained from the activity meter test.

Anxiety studies were performed in the mice models. The amelioration of innate anxiety induced by novel environment was explored in the elevated plus maze test and the regular Suok test. The elevated plus maze and Suok tests assess the behaviour of mice in a conflict situation. The elevated plus maze assesses the aversion to height and open spaces [20]. The Suok test, however, possesses a unique advantage of assessing the anxiety state of rodents as well as the sensorimotor coordination on an elevated horizontal rod [16, 21]. In general, anxiolytics are known to increase affinity for the aversive stimuli or environment whilst anxiogenic agents are known to enhance the innate aversion to these stimuli or environment

In the EPM test, vehicle-treated mice exposed to the maze made fewer entries and spent less time in the open unprotected arms compared to the closed arms, a behaviour which is consistent with literature that mice generally avoid open unprotected arenas [22, 23]. Pretreatment with MAE (100-1000 mg/kg ) or diazepam (0.1-1.0 mg/kg) significantly reversed this behaviour at all tested doses suggesting an anxiolytic property of MAE. Although time spent is key indicator of the anxiety state of the test animal, its measurement provided offers a scintilla of information relating to the actual behavioural measures whilst exploring the maze. It is therefore important to assess, describe, and possibly measure the general behavioural repertoire of the animal of interest in such behavioural assays to offer more insights into the general behaviour of the test animals after treatment with drugs of interest.

Over the past 25 years, several workers have developed protocols that allow a comprehensive profiling of behaviour of mice in the elevated-plus maze based on the defensive behaviours exhibited in the test. While the open arm entries as well as % open arm entries have been found to be a consistent measure of anxiety in rodents, the total head-dips (HD) is a measure of exploration. Additionally, the total SAP provides an additional measure of risk assessment of the test organism. Behaviours such as freezing, stretch attend postures, and head-dips are some of the ethological parameters that can give an indication of the anxiety and behavioural state of the test mouse [24].

Mice in general tend to move freely in the closed arms with an increased tendency to freeze in the open arm of the EPM. Anxiolytics reduce this freezing behaviour while a converse occurs after giving an appropriate dose of an anxiogenic agent. Based on the above facts, it was realized that a single ethological parameter could increase or decrease depending on whether it occurs in the open or closed arms of the EPM. Hence the designation, “protected” and “unprotected”, is ascribed to an ethological parameter occurring in the closed or opened arms, respectively [24, 25].

Anxiogenic agents increase the duration and frequency of protected behaviours (head dips), with a corresponding decrease in unprotected ethological behaviours. The converse is true for agents that possess anxiolytic properties in mice. Similar to diazepam, MAE (1000 mg/kg) administration increased the number of head dips. An increase in the number of head dips is an indication of low anxiety states while a decrease indicates high anxiety states [26].

To assess the effect on sensorimotor coordination and further establish the anxiolytic potential of MAE, the regular Suok test was employed. This test combines aspects of the EPM, OFT, and beam walk tests. Behaviours such as head-dips, side-looks, and frequency and duration of freezing bouts are used as endpoints for assessing of the anxiety state of mice whilst the number of falls and missteps are known to predict the degree of impairment of sensorimotor coordination. Diazepam is known to exhibit anxiolytic effects at lower doses in several test paradigms including the Suok test [16]. However, at relatively higher doses, diazepam induces a state of impaired motor coordination. This made it an ideal positive control in the Suok test which explores both behaviours. MAE (300 mg/kg ) similar to diazepam (0.1-1.0 mg/kg) reduced the number of freezing events suggestive of an anxiolytic effect since increased freezing bouts are indicative of a heightened anxiety state. The sensorimotor coordination was impaired at the highest dose of diazepam (1 mg/kg) which was reflected in the increased number of leg slips. However, the anxiolytic doses of MAE (300 mg/kg) did not affect the number of leg slips. Taken together, it is suggested that MAE exhibits anxiolytic behaviour at doses that does not affect motor coordination although further tests will be required to corroborate this evidence.

Anxiety and depression are intimately linked and usually appear as comorbid states and treatment of both states positively affect the outcome of therapy [27]. Selective serotonin reuptake inhibitors are usually considered first-line treatment for patients with depression and have significant anxiolytic effects [3]. Several classes of drugs that modify serotonin (5-HT) neurotransmission have previously been explored for their possible role in depression and schizophrenia [28]. Based on the above premise, the potential antidepressant effect of MAE was assessed in two acute depression models in mice: tail suspension and forced swim test. These models work on the principle that when mice are subjected to unavoidable, inescapable stress, they assume escape oriented behaviours with intermittent moments of despair usually in the form of immobility [29]. Periods of immobility is known to model some aspects of depressive symptoms and hence most antidepressants are known to decrease the duration of immobility. Consequently, these tests have been employed in the screening of potential antidepressant drugs.

In the TST, significant decrease in immobility duration was achieved after MAE (1000 mg/kg), imipramine (100 mg/kg), and fluoxetine (3-30 mg/kg) treatment suggesting antidepressant activity. Antidepressants that inhibit serotonin and/or NA reuptake decrease immobility and increase swinging behaviour of mice in the TST, a behaviour that was not significantly altered in MAE-treated mice. Opioids are known to decrease immobility whilst increasing curling behaviour [30, 31]. Hence significant increase in the curling duration after MAE administration can be attributed to a possible interaction with the *μ*-opioid receptors.

Similarly, MAE (300 mg/kg) exhibited antidepressant activity comparable to fluoxetine (10 and 30 mg/kg) and imipramine (100 mg/kg) in the FST (Figure 15). Antidepressants acting through the serotonergic system, including fluoxetine, selectively increase swimming behaviour. In addition, the FST differentiates between antidepressants that work through serotonergic mechanisms or noradrenergic mechanisms, as noradrenergic compounds selectively increase climbing behavior [32] and drugs with dual effects increased both swimming and climbing [33]. In this study, MAE caused a dose-dependent reduction in the immobility time at 300 mg/kg, increase in the swimming behaviour, and increase climbing duration at 30-100 mg/kg. This behavioural profile may suggest that the mechanism of the antidepressant-like activity of MAE may be due to an interaction with both noradrenergic and serotonergic system.

## 5. Conclusions

Results from this study indicate that the petroleum ether/ethyl acetate fraction of* Maerua angolensis *stem bark possesses anxiolytic effects in male ICR mice. MAE also possesses antidepressant effects which might be due to interaction with opioid receptors and noradrenergic and serotonergic systems.

## Figures and Tables

**Figure 16:**
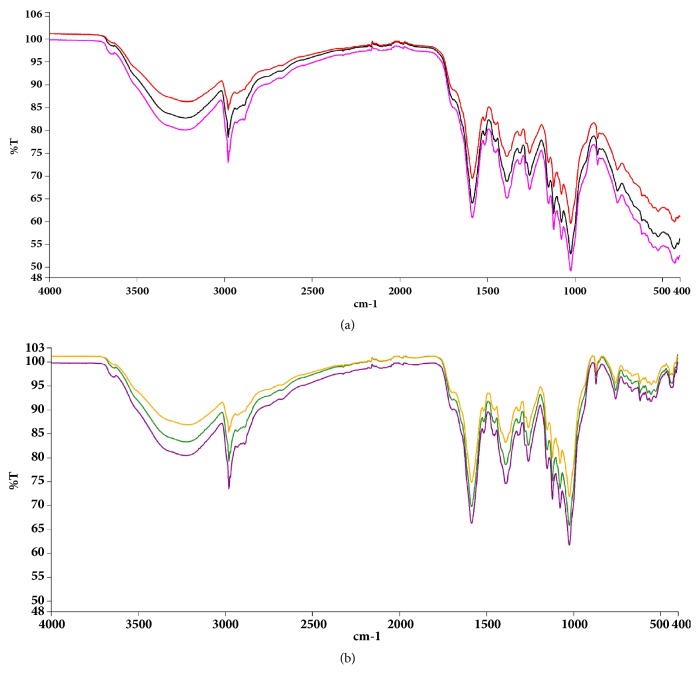
Original spectra of the petroleum ether/ethyl acetate fraction of* Maerua angolensis *stem bark (baseline uncorrected* versus *baseline corrected).

**Table 1:** Effects of *Maerua angolensis* stem bark extract in the Irwin's test.

**Dose (mg kg** ^**-1**^ **)**	**Effects (time period)**	**D/T**

0	No effect (0-180 min)	0/5
100	Analgesia, reduced fear response (60-120 min)	0/5
300	Analgesia, reduced fear response (30-120 min)	0/5
1000	Analgesia, reduced fear and touch response (30-120 min)	0/5
3000	Analgesia, reduced fear, touch response & activity (30-120 min)	0/5
5000	Analgesia, reduced fear, touch response and activity (30-120 min) Increased jumps (15-30 min).	0/5

D= number of deaths recorded T= number of animals treated

**(a):** 

Peak	X (cm-1)	Y (%T)	Peak	X (cm-1)	Y (%T)	Peak	X (cm-1)	Y (%T)	Peak	X (cm-1)	Y (%T)
1	3226.52	82.82	2	2980.94	78.51	3	2100.15	98.53	4	1585.85	64.02

5	1515.39	78.99	6	1454.94	77.64	7	1386.52	68.83	8	1310.96	75.08

9	1258.88	70.16	10	1150.77	67.59	11	1120.87	61.74	12	1076.32	59.83

13	1023.17	52.81	14	869.99	74.68	15	755.57	66.71	16	523.8	56.6

17	430.93	53.96									

**(b):** 

Peak	X (cm-1) (cm-1)	Y (%T) (%T)	Peak	X (cm-1) (cm-1)	Y (%T) (%T)	Peak	X (cm-1) (cm-1)	Y (%T) (%T)	Peak	X (cm-1) (cm-1)	Y (%T) (%T)
1	3224.35	86.36	2	2980.96	84.61	3	2099.5	98.76	4	1585.79	69.48

5	1515.51	82.17	6	1454.78	81.33	7	1386.55	74.32	8	1310.69	78.91

9	1258.95	74.98	10	1150.65	73.01	11	1120.85	67.62	12	1076.39	66

13	1022.95	59.51	14	869.99	78.29	15	755.56	71.36	16	523.2	62.18

17	430.37	59.83									

**(c):** 

Peak	X (cm-1) (cm-1) (cm-1)	Y (%T) (%T) (%T)	Peak	X (cm-1) (cm-1) (cm-1)	Y (%T) (%T) (%T)	Peak	X (cm-1) (cm-1) (cm-1)	Y (%T) (%T) (%T)	Peak	X (cm-1) (cm-1) (cm-1)	Y (%T) (%T) (%T)
1	3227.3	80.11	2	2980.92	73.02	3	2103.82	97.56	4	1585.89	60.85

5	1515.37	76.86	6	1455	75.17	7	1386.39	65.15	8	1311.06	72.64

9	1258.82	67.03	10	1150.79	63.95	11	1120.87	58.23	12	1076.26	56.09

13	1023.43	49.11	14	869.93	72.48	15	755.56	64.08	16	523.45	53.54

17	427.16	50.8	18	407.14	51.61						

## Data Availability

The data used to support the findings of this study are available from the corresponding author upon request.

## Appendix

See Table 2 and Figure 16.

## Abbreviations

ANOVA: Analysis of variance
CNS: Central nervous system
Dzp: Diazepam
EPM: Elevated-plus maze
FT-IR: Fourier transform infrared spectroscopy
FST: Forced swim test
HD: Head dips
i.p.: Intraperitoneal
IR: Infrared spectroscopy
KNUST: Kwame Nkrumah University of Science and Technology
MAE: * Maerua angolensis* extract
OFT: Open field test
PTZ: Pentylenetetrazole
pHD: Protected head-dips
*p.o.*: * Per os*
SAP: Stretch attend posture
*s.c.*: Subcutaneous
SEM: Standard error of mean
TST: Tail suspension test.

## Additional Points

*Chemical Compounds Studied in this Article*. Diazepam (PubChem CID: 3016), Fluoxetine (PubChem CID: 3386), Imipramine (PubChem CID: 3696), Caffeine (PubChem CID: 2519), Pentobarbitone sodium (PubChem CID: 23676152), and Pentylenetetrazole (PubChem CID: 5917).

## References

[1] Ohayon M. M. (2007). Epidemiology of depression and its treatment in the general population. *Journal of Psychiatric Research*.

[2] Patel V., Araya R., Bolton P. (2004). Editorial: Treating depression in the developing world. *Tropical Medicine & International Health*.

[3] Pollack M. H. (2005). Comorbid anxiety and depression. *Journal of Clinical Psychiatry*.

[4] Adongo D. W., Kukuia K. K. E., Mante P. K., Ameyaw E. O., Woode E. (2015). Antidepressant-like effect of the leaves of Pseudospondias microcarpa in mice: evidence for the involvement of the serotoninergic system, NMDA receptor complex, and nitric oxide pathway. *BioMed Research International*.

[5] Adongo D. W., Mante P. K., Kukuia K. K. E. (2017). Anticonvulsant activity of Pseudospondias microcarpa (A. Rich) Engl. hydroethanolic leaf extract in mice: The role of excitatory/inhibitory neurotransmission and nitric oxide pathway. *Journal of Ethnopharmacology*.

[6] Biney R. P., Benneh C. K., Ameyaw E. O., Boakye-Gyasi E., Woode E. (2016). Xylopia aethiopica fruit extract exhibits antidepressant-like effect via interaction with serotonergic neurotransmission in mice. *Journal of Ethnopharmacology*.

[7] Chhabra S. C., Mahunnah R. L., Mshiu E. N. (1989). Plants used in traditional medicine in Eastern Tanzania. II. Angiosperms (capparidaceae to ebenaceae). *Journal of Ethnopharmacology*.

[8] Azi Iliya H., Kofi W., Abotsi M., Benneh C., Woode E. (2016). Maerua angolensis extract reduces allodynia and hyperalgesia in a mouse model of vincristine-induced peripheral neuropathy. *Journal of Applied Pharmaceutical Science*.

[9] Magaji M. G., Yaro A. H., Adamu A. (2009). Some neuropharmacological studies on hydroalcoholic extract of Maerua angolensis DC (Caparidaceae) in mice and chicks. *International Journal of Pure and Applied Sciences*.

[10] Malami I., Hassan S. W., Alhassan A. M., Shinkafi T. S., Umar A. T., Shehu S. (2014). Anxiolytic, sedative and toxicological effect of hydromethanolic stem bark extract of Maerua angolensis DC. in Wister rats. *Pakistan Journal of Pharmaceutical Sciences*.

[11] Meda N. T. R., Bangou M. J., Bakasso S., Millogo-Rasolodimby J., Nacoulma O. G. (2013). Antioxidant activity of phenolic and flavonoid fractions of Cleome gynandra and Maerua angolensis of Burkina faso. *Journal of Applied Pharmaceutical Science*.

[12] Azi I. H., Eric B.-G., Donatus A. W., Agyei A. F., Eric W. (2014). Antinociceptive activity of various solvent extracts of Maerua angolensis DC stem bark in rodents. *The Journal of phytopharmacology*.

[13] Benneh C. K., Biney R. P., Mante P. K., Tandoh A., Adongo D. W., Woode E. (2017). Maerua angolensis stem bark extract reverses anxiety and related behaviours in zebrafish—Involvement of GABAergic and 5-HT systems. *Journal of Ethnopharmacology*.

[14] Benneh C. K., Biney R. P., Tandoh A. (2018). *Maerua angolensis* DC. (capparaceae) stem bark extract protects against pentylenetetrazole-induced oxidative stress and seizures in rats. *Evidence-Based Complementary and Alternative Medicine*.

[15] Irwin S. (1968). Comprehensive observational assessment: Ia. A systematic, quantitative procedure for assessing the behavioral and physiologic state of the mouse. *Psychopharmacology*.

[16] Kalueff A. V., Keisala T., Minasyan A. (2008). The regular and light-dark Suok tests of anxiety and sensorimotor integration: Utility for behavioral characterization in laboratory rodents. *Nature Protocols*.

[17] Pawlak C. R., Karrenbauer B. D., Schneider P., Ho Y.-J. (2012). The elevated plus-maze test: Differential psychopharmacology of anxiety-related behavior. *Emotion Review*.

[18] Porsolt R. D., Bertin A., Jalfre M. (1977). Behavioral despair in mice: a primary screening test for antidepressants. *Archives Internationales de Pharmacodynamie et de Thérapie*.

[19] Essman W. B. (1983). Serotonin in learning and memory. *Clinical Pharmacology of Learning and Memory*.

[20] Bourin M., Petit-Demoulière B., Nic Dhonnchadha B., Hascöet M. (2007). Animal models of anxiety in mice. *Fundamental & Clinical Pharmacology*.

[21] Kalueff A. V., Tuohimaa P. (2005). The Suok ("ropewalking") murine test of anxiety. *Brain Research Protocols*.

[22] Carobrez A. P., Bertoglio L. J. (2005). Ethological and temporal analyses of anxiety-like behavior: the elevated plus-maze model 20 years on. *Neuroscience & Biobehavioral Reviews*.

[23] Lister R. G. (1987). The use of a plus-maze to measure anxiety in the mouse. *Psychopharmacology*.

[24] Rodgers R. J., Cao B.-J., Dalvi A., Holmes A. (1997). Animal models of anxiety: An ethological perspective. *Brazilian Journal of Medical and Biological Research*.

[25] Cole J. C., Rodgers R. J. (1994). Ethological evaluation of the effects of acute and chronic buspirone treatment in the murine elevated plus-maze test: comparison with haloperidol. *Psychopharmacology*.

[26] Rodgers R. J., Johnson N. J. T. (1995). Factor analysis of spatiotemporal and ethological measures in the murine elevated plus-maze test of anxiety. *Pharmacology Biochemistry & Behavior*.

[27] Outhoff K. (2010). The pharmacology of anxiolytics. *South African Family Practice*.

[28] Levy A. D., Van de Kar L. D. (1992). Endocrine and receptor pharmacology of serotonergic anxiolytics, antipsychotics and antidepressants. *Life Sciences*.

[29] Steru L., Chermat R., Thierry B., Simon P. (1985). The tail suspension test: a new method for screening antidepressants in mice. *Psychopharmacology*.

[30] Berrocoso E., Ikeda K., Sora I., Uhl G. R., Sánchez-Blázquez P., Mico J. A. (2013). Active behaviours produced by antidepressants and opioids in the mouse tail suspension test. *The International Journal of Neuropsychopharmacology*.

[31] Berrocoso E., Mico J.-A. (2009). Cooperative opioid and serotonergic mechanisms generate superior antidepressant-like effects in a mice model of depression. *The International Journal of Neuropsychopharmacology*.

[32] Detke M. J., Rickels M., Lucki I. (1995). Active behaviors in the rat forced swimming test differentially produced by serotonergic and noradrenergic antidepressants. *Psychopharmacology*.

[33] Rénéric J.-P., Lucki I. (1998). Antidepressant behavioral effects by dual inhibition of monoamine reuptake in the rat forced swimming test. *Psychopharmacology*.

